# Determinants of Quality of Life and Treatment Satisfaction During Long-Term Involuntary In-patient Treatment of Dual-Diagnosis Patients

**DOI:** 10.3389/fpsyt.2022.801826

**Published:** 2022-02-10

**Authors:** Grieke D. Van Kranenburg, Wout J. Diekman, Rob H. S. Van den Brink, Wijnand G. Mulder, G. H. M. Pijnenborg, C. L. Mulder

**Affiliations:** ^1^Mental Health Organization Drenthe, Sustainable Residence, Beilen, Netherlands; ^2^Addiction Service North Netherlands, Groningen, Netherlands; ^3^Department of Psychiatry, Rob Giel Research Center, University Medical Center Groningen, University of Groningen, Groningen, Netherlands; ^4^Fivoor Mental Health Organization, The Hague, Netherlands; ^5^Department of Psychotic Disorders, Drenthe Mental Healthcare Organization, Assen, Netherlands; ^6^Department of Clinical Psychology and Experimental Psychopathology, Faculty of Behavioural and Social Science, University of Groningen, Groningen, Netherlands; ^7^Department of Psychiatry, Erasmus Medical Center, Epidemiological and Social Psychiatric Research Institute, Rotterdam, Netherlands

**Keywords:** involuntary hospital admission, quality of life, treatment satisfaction, severely mentally ill, dual diagnosis, difficult-to-engage

## Abstract

**Introduction:**

Treatment resistance and disengagement from mental health services are major obstacles in the treatment of dual diagnosis patients with Severe Mental Illness. The patients in this study were admitted to a long-term involuntary treatment facility.

**Aim of the study:**

To study which patient experiences and perceptions are related to the outcome measures Subjective Quality of Life (SQOL) and Treatment Satisfaction (TS) during the long-term involuntary treatment.

**Methods:**

Patients were invited for an interview by an independent researcher, which included self-report questionnaires. The structured interviews included self-assessing Helping Alliance, Insight, Attitude toward involuntary admission, Perceived coercion and Perceived benefit were studied as determinants of SQOL and TS. The relationship between the determinants and the outcomes were analyzed by linear regression analysis.

**Results:**

Patient reported outcomes from dual diagnosis patients in a long-term treatment facility, showed that most of the patients, in spite of the involuntary character of the treatment, were satisfied with the treatment. With respect to the determinants of SQOL and TS the perceptions that “My opinion is taken into account” and “Perceived benefits of the treatment” are strong predictors of both the outcomes.

**Conclusions:**

The current study shows that the most important aspects for treatment satisfaction and quality of life of dual-diagnosis patients admitted involuntary to long-term treatment, are being listened to (being taken seriously) and experiencing improvements during treatment. These qualities reflect the goals of Shared Decision Making and Perceived Procedural Justice in treatment. The study also corroborates earlier findings that even when treated involuntarily, patients might not hold particular negative views regarding their treatment.

## Introduction

Treatment resistance and disengagement from mental-health services are major obstacles in the treatment of dual diagnosis patients with Severe Mental Illness (SMI) and substance use disorder. About 50% of these patients do not respond well to integrated outpatient services (1), in part, because they lack stable, safe and supportive living arrangements.

There is evidence that long-term residential dual-diagnosis programs can be effective for dual- diagnosis patients who did not respond to outpatient treatment (2). However, when these programs are voluntary, their attrition rate can be as high as 75% (1). Long term compulsory treatment can be an option for patients who need mental healthcare and pose a severe risk to themselves or others, but continuously drop out of voluntary programs.

The patients in this study were admitted to a long-term compulsory treatment facility, based on a Dutch civil law court order. To obtain such order, an independent psychiatrist makes an assessment which is requested by the treatment provider. The assessment is sent to the judge, who decides to such an order or to an extension of the order every 6 or 12 month. The patients were at high risk of ultimate self-neglect and societal deterioration, and had been treated by all available means—including frequent compulsory hospital-admissions—without lasting improvements.

Because of the long-term and compulsory character, evaluation of the treatment is important from both a clinical and an ethical point of view: the treatment is seen as an “ultimum remedium” and the effects of the long-term compulsory treatment are unknown so far. Restricting patients autonomy over a long period of time seems at odds with the reforms in mental healthcare and is therefore controversial. Hence, evaluation of treatment effects is needed to indicate whether this type of compulsory impatient treatment benefits patients.

In a previous article (3) the clinical and functional outcomes of the treatment were reported. This article concerns the patient reported outcomes (PRO's) which are an essential part of the evaluation of the treatment.

Given the involuntary nature of the treatment in this study Treatment Satisfaction (TS) is an important measure since patients cannot discontinue their treatment when displeased with it. In addition more Treatment Satisfaction is associated with better clinical outcomes (4). Because of the long duration of the treatment, patients live in the clinic for a long time which makes their Subjective Quality of Life (SQOL) an issue of serious concern. Studies on homelessness show that having a house or somewhere to sleep where you feel reasonably safe is related to better quality of life (5, 6). However, these studies did not concern patients who were involuntarily committed to treatment.

Research into patients' views on their involuntary hospitalization was done in the InvolvE (7) and Eunomia (8) studies, two large European studies assessing outcomes of involuntary psychiatric inpatient treatment. Predictors of the outcomes were also studied and included the patient's perceived coercion, illness insight, experienced therapeutic relationship, feeling of justification of involuntary admission, and perceived benefits from inpatient treatment. An important conclusion from these studies was that even when patients are treated involuntary, patients might not hold particularly negative views regarding treatment (9). The concept of “Perceived procedural justice” (9) emphasizes the importance of how patients feel they are being treated during their hospitalization. This seems even more important than whether their treatment is voluntary or involuntary. Perceived procedural justice represents the patient's perception that others are acting out of genuine concern for them and that they are being listened to and treated with respect and fairness. The level of perceived procedural justice is positively correlated with TS (4).

The above studies concerned short-term involuntary treatment and had a retrospective character.

Here we aim to investigate which patient experiences and perceptions are related to the outcome measures Subjective Quality of Life and Treatment Satisfaction during long-term compulsory treatment in an inpatient setting. Identifying the determinants of Subjective Quality of Life and of Treatment Satisfaction may offer suggestions to improve these treatment outcomes and hence the experience of being committed to long-term compulsory treatment.

## Methods

### Patients and Setting

Earlier this century the Dutch Government decided to establish a unique purpose-built long-term compulsory treatment facility, called Sustainable Residence (SuRe).

This new treatment facility was intended for homeless dual- diagnosis patients whom existing services considered to be treatment-resistant. Target population are dual diagnosis patients at high risk of ultimate self-neglect and societal deterioration, who cause considerable public nuisance. The patients have a long history of treatment (including multiple compulsory admissions) which did not lead to lasting improvements.

Patients can be hospitalized for as long as necessary on the basis of a court order determined by an independent psychiatrist and a civil law judge, the latter deciding on extension every 6 or 12 months. Treatment in SuRe is aimed at improving patients' quality of life and functioning to a level necessary for living in a less restrictive and less supportive environment.

Patients are involuntary admitted to SuRe and consequently the area is closed by a fence. After the first 2 weeks of admission patients have permission to (escorted) leave SuRe, mostly every day on agreed times. Additionally the only general obligation is to participate in alcohol and drug checks when entering SuRe. There is no other general form of compulsory care.

There are four criteria for admission to SuRe: (1) dual diagnosis (i.e. SMI and substance use disorder); (2) a history of homelessness; (3) failure of earlier treatment to achieve lasting improvement, despite the use of appropriate means, including multiple compulsory admissions and (4) the imposition of a civil-law court order for involuntary admission on the grounds of the risk of lasting danger to self or others. The main criterion for discharge from SuRe is a sufficiently reduced risk to oneself and/or others which is necessary for being discharged to a less restricted and less supportive setting.

To our knowledge, SuRe is the only treatment facility worldwide, in which dual-diagnosis patients are admitted for long-term compulsory treatment based on a civil court order.

### Treatment

The treatment at SuRe, which is comprehensive and highly supportive, is delivered by nine multidisciplinary teams consisting of a psychiatrist, psychologist, case managers, residential supervisors, and domestic workers. Other disciplines such as a physician, nurses, social workers, creative therapists, psychomotor therapists, social juridical workers, activity supervisors, and a cultural anthropologist are also available. The treatment at SuRe is based on the principles of recovery: patient-centered, and focused on offering hope and perspective. All patients have a room or house in a closed area that was designed according the principles of a “healing environment.” This concept implies that the physical healthcare environment can make a difference in how quickly the patient recovers from or adapts to specific acute and chronic conditions.

The facility also has a crisis unit and a small unit for long, intensive care. Sure has a maximum capacity of 133 patients.

### Study Design

For this study all patients who were in treatment at SuRe between January 2010 and November 2012 were invited for an interview by an independent researcher, which included self-report questionnaires. Because patients admitted to SuRe can be disorganized or have problems with concentration, we chose to conduct the questionnaires by interview, in which we read aloud the questions and answering verbatim. In this way we could check whether the patient understood the information and, if necessary, could elucidate it a bit more. Patients were interviewed yearly during the study period. For this study we used patients' first interview after admission.

The study protocol was reviewed and approved by the Dutch Medical Ethical Committee for the Mental Health Services and judged to be in accordance with the Dutch Medical Research Involving Human Subjects Act. (Metc nr: NL30019.097.09).

### Instruments

#### Outcome Measures

Treatment Satisfaction (TS) and Subjective Quality of Life (SQOL) are important PRO's of mental healthcare and are often part of the Routine Outcome Assessments of treatment. Although Quality of Life and Treatment Satisfaction are strongly associated, they can provide distinct information independent from overlap (10).

##### Treatment Satisfaction

Patients' appraisal of the inpatient treatment was assessed with the Client's Assessment of Treatment scale (CAT) (11) which comprises seven items (i.e., “Do you believe you are receiving the right treatment for you?” “Does your psychiatrist understand you and is he/she engaged in your treatment?” “Are relations with other staff members pleasant for you?” “Do you believe you are receiving the right medication for you?” “Do you believe the other elements of treatment are right for you?” “Do you feel respected and regarded well?” and “Has treatment been helpful for you?”). Each item is rated on a scale from 0 (not at all) to 10 (yes entirely). The mean score of all items was used as outcome measure. Higher scores indicate more satisfaction with treatment. The CAT has been widely used with psychiatric inpatients, has good internal consistency and demonstrates good factorial validity and invariance (12).

##### Subjective Quality of Life

To assess subjective quality of life the Manchester Short Assessment of Quality of Life (MANSA) (13) was used. This instrument consists of twelve items regarding satisfaction with different aspects of life and life as a whole. The items are rated on seven-point Likert scales (1 = could not be worse, 7 = could not be better; mean score of all items used). The question about job satisfaction was excluded because none of the patients had a paid job during admission. A high score indicates a high quality of life. The MANSA has good validity and reliability. Besides the above questions on satisfaction, the MANSA contains four factual yes/no questions which are disregarded here.

#### Determinants

The following variables are studied as determinants of SQOL and TS: Helping alliance, Insight, Attitude toward involuntary admission, Perceived coercion, and Perceived benefit.

##### Helping Alliance

The patient's perception of the quality of the therapeutic alliance with treatment providers was assessed using the client version of the Helping Alliance Scale (HAS) (14). This scale includes five items covering basic elements of a therapeutic relationship, such as the extent to which the patient feels understood by his or her clinician and how much the patient's treatment reflects mutually agreed goals. These items are rated on 10-point scales. A sum score of the five items is calculated, a higher score indicates a better therapeutic relationship. Patients were asked to name the case manager they felt was most involved in their treatment and to answer the HAS items for their relationship with this person. In the analyses of the relationship of the HAS with the TS outcome, the first item of the HAS was omitted because it is identical to the first item of the CAT.

##### Insight

Level of insight into illness was measured using the Birchwood Insight Scale (BIS) (15). This is an eight-item self-report questionnaire consisting of three dimensions relating to patient insight: “relabelling of symptoms” (i.e., denying their pathological nature; sum of items 1 and 8), “awareness of mental illness” (sum of items 2 and 7) and “recognition of a need for treatment” (sum of items 3, 4, 5, and 6 divided by 2) and a total insight score (sum of all items). Each item is rated as “agree,” “disagree,” or “unsure,” giving an item score of 1 for unsure, and 0 or 2 for agree and disagree depending on whether agreement with the statement indicates good insight (the items are counterbalanced for response valence). For this study in dual diagnosis patients we used two versions of item 7 of the BIS: the original version on awareness of “mental illness” and—because we studied dual diagnosis patients—we added a second version inquiring about awareness of “an addiction problem.” We took the mean score of both as score on item 7. An outcome of 3 or more on a subscale and 9 or more on the total scale indicates good insight.

##### Attitude Toward Involuntary Admission

Three questions assessing the attitude toward involuntary admission were derived from the InvolvE study (7). The first assesses “Justification of admission” by the question: “Today, do you find it right or wrong that you were admitted to the hospital?” Responses could be rated on a scale from 0 (entirely wrong) to 10 (entirely right) and were later dichotomised as un-justified (0–5) and justified (6–10) to indicate a generally negative or positive attitude toward the involuntary admission.

The second question assessed “perceived risk to self,” and read “Do you think you posed a risk to yourself when you were admitted to SuRe under the Mental Health Act?” The third question assessed “perceived risk to others” by “Do you think you posed a risk to others when you were admitted to SuRe under the Mental Health Act?” Responses to these latter questions were rated as 0 “no” and 1 “yes.”

##### Perceived Coercion During Treatment

To assess perceived coercion, the following three questions, based on the McArthur Perceived Coercion Scale (16):

“I feel free to participate or not in the treatment,” “As for treatment my opinion is taken into account,” and “I decide whether or not to take medication.”

Response options for these questions were “agree,” “unsure,” and “disagree,” which were dichotomized into (1) agree vs. (0) “unsure” or “disagree.” Higher scores indicate less perceived coercion.

##### Perceived Benefit

Perceived benefits from the inpatient treatment was assessed by the question: “With regard to your mental health and addiction problems, how do you feel now in comparison to when you were admitted?” This question was derived from the InvolvE study (17) (Katsakou, Personal Communication). Responses could be rated on a scale from 0 (much worse) to 10 (much better). A score of 6 or more was taken to indicate perceived improvement of mental health and addiction problems.

### Analysis

Descriptive statistics were used to depict the scores on the outcomes and determinants. Patients who participated in the study were compared to those who did not, on their demographic and clinical characteristics. Subsequently the relationships between the determinants and outcomes were analyzed by linear regression analysis. This was carried out for each outcome separately and in two steps. First the association between individual determinants and outcomes listed above was examined by univariate linear regression analysis. Second, to explore which variables were independent determinants of the outcome variable, we performed a multivariate linear regression analysis including all determinants with a significant association at an alpha level of 0.05 or less in the univariate analyses. The goodness of fit of the univariate and multivariate models was evaluated by the proportion of variance of the outcome variable explained by the determinants included in the model; i.e., by the Beta-square for the univariate models and the R-square statistic for the multivariate model including the significant determinants only. Both in the univariate and multivariate models, the Beta statistic of each determinant is an effect size measure for the strength of the association between that determinant and the outcome variable. The absence of multicollinearity in the multivariate models was checked by testing for all determinants whether the tolerance (i.e., the proportion of variance of the determinant not explained by the other determinants included in the model) was 0.20 or more.

The time of assessment after admission varied widely between patients. All linear regression analyses were therefore controlled for time of assessment after admission to SuRe.

## Results

During the study period 156 patients were treated in SuRe. Fourteen patients did not want to participate in the study interview and fourteen could not participate for several reasons (e.g., being discharged before an interview was arranged or because of psychological problems). In Table 1 the demographic and clinical characteristics of the participants and non-participants are compared.

**Table 1:** Demographic and clinical characteristics of dual diagnosis patients admitted to SuRe.

	**Study sample** **(*N* = 128)**	**Non** **respondents** **(*N* = 28)**	**X^**2**^/T**	***p***
Gender (% male)	79.7	82.1	0.087	0.77
Age (mean in years, sd)	39.9 (8.5)	38.9 (7.9)	0.567	0.57
**Education**^&^ **(%**^#^**)**				
Low	62.2	76.2	1.953	0.38
Intermediate	28.8	14.3		
High	9.0	9.5		
Missing	13.3	25.0		
**Diagnosis on axis I (%** ^#^ **)**				
Psychotic disorder	89.8	92.0	0.110	0.74
Substance abuse	93.8	92.0	0.105	0.75
Missing	0	10.7		
**Diagnosis on axis II (%** ^#^ **)**				
Personality disorder	40.5	50.0	0.674	0.41
Borderline intellectual functioning	18.9	13.6	0.346	0.56
Missing	13.3	21.4		
Duration of admission at time of interview (mean in days, sd)	403 (419)	N/A	N/A	N/A

& *Low: elementary school or less; intermediate: low-level/intermediate level secondary school; high: high level secondary school, intermediate vocational, or higher education*.

# *Valid percentages (i.e., when missing data are excluded)*.

In terms of age, sex, education and diagnosis, non-respondents did not differ from respondents.

The study sample was predominantly male and represented a wide age range (from 22 to 59 years). Upon referral to SuRe patients had, almost without exception, been diagnosed with a psychotic disorder, particularly paranoid schizophrenia (58.2%) and disorganized schizophrenia (15.0%). In addition, almost all had a substance use or dependence disorder, usually involving multiple drugs. A substantial proportion of the patients had borderline intellectual functioning or less (defined as an IQ < 85). Over half, the patients had a low educational level (elementary school or less).

Table 2 presents the distribution of assessments of Treatment Satisfaction, Subjective Quality of Life and the determinants. With respect to Treatment Satisfaction, 51.4% had a mean score of 7 or more which can be taken to indicate they are reasonably to very satisfied with the treatment. 34.5% of the patients had a mean score of 5 or lower indicating dissatisfaction with the treatment. Concerning Quality of Life 5.4% had a mean score below 3 which was labeled as “very dissatisfied” or worse and 26.8% a score of 5 or more, which referred to “reasonably satisfied” or better. The largest group (67.8%) rated between 3 and 5. The determinant Insight shows that 31.2% had good insight on the aspect of “Relabeling of symptoms,” 10.9% on “Awareness of mental illness,” 48.6% on “Recognition of a need for treatment,” and 17.3% on the Total insight scale. Almost 4 out of 10 patients judged their involuntary admission as right. Most of the patients (76.0%) experienced improvements of their mental health problems.

**Table 2:** Distribution of treatment satisfaction, subjective quality of life and determinants assessed.

	***N***	**Mean (SD)/** **percentage**
Satisfaction with treatment (CAT)	107	6.18 (3.00)
Subjective quality of life (MANSA)	112	4.50 (0.90)
Helping alliance (HAS-client version)	91	7.11 (2.87)
Insight (BIS-total score)	99	5.32 (3.15)
Relabeling of symptoms	106	1.70 (1.40)
Awareness of mental illness	120	1.33 (1.24)
Recognition of a need for treatment	111	2.29 (1.44)
**Attitude toward involuntary admission**		
Justification of admission (% justified)	116	39,7%
Risk to self (% yes)	118	22.9%
Risk to others (% yes)	116	15.5%
**Perceived coercion during treatment**		
I feel free to participate or not in the treatment (% agree)	117	60.7%
As for treatment my opinion is taken into account (% agree)	120	58.3%
I decide whether or not to take medication (% agree)	119	41.2%
Perceived benefits from inpatient treatment	104	7.27 (3.27)

Table 3 presents the associations of the determinants with Treatment Satisfaction. The table shows that all determinants have aspects which are significantly associated with Treatment Satisfaction in univariate analyses. For Insight the aspect “Relabeling of symptoms” was not significantly related. The same is found for the aspects “Perceived risk to self” and “Perceived risk to others,” which are part of the determinant “Attitude toward involuntary admission.”

**Table 3:** Determinants of treatment satisfaction.

	**Univariate**		**Multivariate**	
	***Beta***	***P***	***Beta***	***P***
Helping alliance (HAS)	0.221	0.06	N/A	
**Insight (BIS)**				
Relabeling of symptoms	0.060	0.57	N/A	
Awareness of mental illness	0.229	0.02	−0.016	0.85
Recognition of a need for treatment	0.562	<0.01	0.347	<0.01
**Attitude toward involuntary admission**				
Justification of admission	0.565	<0.01	0.237	0.01
Perceived risk to self	0.002	0.98	N/A	
Perceived risk to others	0.132	0.20	N/A	
**Perceived coercion during treatment**				
I feel free to participate in treatment	0.334	<0.01	0.101	0.20
My opinion is taken into account	0.472	<0.01	0.247	<0.01
I decide whether or not to take medication	0.202	0.04	0.146	0.06
Perceived benefits from inpatient treatment	0.436	<0.01	0.213	0.02

The multivariate analysis shows that four variables prove to be independent determinants of Treatment Satisfaction. These are (in order of effect size): “Recognition of need for treatment,” “My opinion is taken into account,” “Justification of admission,” and “Perceived benefits from inpatient treatment.” These four determinants together explain 55% of the variance in Treatment Satisfaction. The minimal tolerance of the determinants was 69% indicating that there was no multicollinearity between the determinants.

Table 4 presents the associations of the determinants with the Subjective Quality of Life. It shows that all determinants have aspects which are significantly related to Subjective Quality of Life in univariate analyses, with the exception of the Helping Alliance. Recognition of a need for treatment, the feeling that the admission was justified, that the treatment is beneficial, feeling free to participate in treatment and that there is consideration for one's opinion are positively related to SQOL.

**Table 4:** Determinants of subjective quality of life.

	**Univariate**		**Multivariate**	
	***Beta***	***P***	***Beta***	***P***
Helping alliance (HAS	0.137	0.24	N/A	
**Insight (BIS)**				
Relabeling of symptoms	−0.077	0.45	N/A	
Awareness of mental illness	−0.038	0.70	N/A	
Recognition of a need for treatment	0.263	<0.01	0.160	0.12
**Attitude toward involuntary admission**				
Justification of admission	0.311	<0.01	0.046	0.67
Perceived risk to self	−0.109	0.27	N/A	
Perceived risk to others	0.030	0.77	N/A	
**Perceived coercion during treatment**				
I feel free to participate in treatment	0.257	<0.01	0.109	0.27
My opinion is taken into account	0.416	<0.01	0.307	<0.01
I decide whether or not to take medication	−0.038	0.70	N/A	
Perceived benefits from inpatient treatment	0.419	<0.01	0.294	<0.01

Multivariate analysis, however, shows that these determinants overlap to some extent. Only the perceptions of benefit of treatment and consideration of one's opinion prove to be independent determinants of SQOL. Together these two determinants explain 29% of the variance of SQOL and show no multicollinearity with a tolerance of 91% between the determinants.

There are some differences in the associations of the determinants with the outcome variables. In the univariate analyses the variables “Awareness of illness” and “I decide whether or not to take medication” are related to Treatment Satisfaction but not to Quality of Life.

## Discussion

In this study we investigated determinants of treatment satisfaction and quality of life in dual diagnosis patients in a long-term compulsory treatment setting. Patients admitted to SuRe proved to be rather satisfied with their treatment with 51.4% scoring on average 7 or higher on 10-point scales and 34.6% scoring on average a 5 or less indicating dissatisfaction with the treatment. 65.4% had an average score above 5. This is comparable to a fluctuating 58–66% above 5 over the 1 year follow-up period of the InvolvE study on short-term involuntary treatment. With respect to their quality of life, 5% of the patients indicate they were very dissatisfied with their lives as a whole and 27%, on the other hand, that they were reasonably to very satisfied.

These figures show that, in spite of the involuntary character of the treatment, most of the patients were satisfied with the treatment. At the same time: there is room for improvement in treatment satisfaction and especially quality of life of patients admitted to long-term compulsory treatment. Determinants of these patient experiences may provide suggestions how to improve this during the treatment.

With respect to the determinants of treatment satisfaction and quality of life of patients admitted to a long-term compulsory treatment, we found that the feeling that one's opinion is taken into account and that the treatment is beneficial are strong independent predictors of both patient outcomes. This suggest that participation of patients in treatment and sincerely listening to and considering the patient's opinion on treatment decisions and treatment effects, may contribute to a more positive experience of being admitted compulsory. This is in line with the results of studies on the effects of Shared Decision Making (SDM) in somatic and mental healthcare in general, which show that SDM is positively related to cognitive-affective outcomes of treatment, such as patients satisfaction, but not distinctly to behavioral and health outcomes such as quality of life (18). That we in contrast did find a positive relationship between feeling one's opinion is taken into account and the quality of life of the patients, may result from the long-term commitment of the patients in our study to the treatment facility, whereas most SDM studies were conducted in an outpatient or short-term setting (18). It may be worthwhile to take this difference in treatment setting into account in studies on the effect of SDM on the quality of life of the patients.

For treatment satisfaction we found two additional independent predictors: “Recognition of a need for treatment” and “Justification of admission.” Although the patients of SuRe have a relatively poor illness insight (overall 17.3% had good insight) compared to 48% in a study by Tait et al. (19) and 50.7% in a study by Quee et al. (20), recognition of the need for treatment proves to be the strongest determinant of treatment satisfaction in our study, and about half of the patients scored well on this aspect of illness insight. This high number is remarkable, because in the EUNOMIA study it is reported that the diagnosis schizophrenia, which is dominant among the patients of SuRe, is associated with a lack of insight, and patients of SuRe are typically care-avoidant and need to be admitted involuntarily. Nevertheless, realizing one's need for treatment appears to be an important prerequisite for experiencing the treatment—although administered compulsory—as satisfying. In addition, “Justification of admission” is another strong determinant of treatment satisfaction and 40% of the patients studied judged their involuntary admission to be justified. This again is at odds with the negative views on admission found in the Eunomia study (21) among patients with schizophrenia. Long term commitment to compulsory treatment may influence patients' view of the necessity and justification of their admission to treatment, and this may be another prerequisite for satisfaction with the treatment received.

The above observations underscore the conclusion of the InvolvE study (7) that “even when treated involuntarily, patients might not hold particularly negative views regarding their treatment.” In this context several authors point to the importance of “Perceived procedural justice” for patient satisfaction in involuntary treatment (9). This concept means that patients should feel that staff treats them respectfully, genuinely cares about their wellbeing and do not restrict their autonomy unnecessary but invite them to participate in treatment decisions. The present study shows that “Perceived procedural justice” is also crucial for a positive effect on patients outcomes of long-term inpatient treatment of compulsory admitted dual-diagnosis patients. For patients who express that they are dissatisfied with treatment or dissatisfied with their life, this would mean that staff should increase their efforts to show they care about these negative feelings and are willing to support these patients and adjust treatment where possible.

In a previous study we showed that the life history of most of the patients committed to the long-term compulsory treatment prove to be extremely troublesome (22). The ambitions for treatment should therefore be realistic and in accordance with these adverse circumstances. In another study we showed, however, that treatment gains can be attained by long-term compulsory inpatient treatment, so that 42% of the patients can be referred to a less restrictive and less supportive setting within 4 years (3). This is supported by the fact that in the current study 76% of the patients indicated that they experienced improvements in mental health during their treatment.

## Limitations

Several limitations of the current study need to be addressed. First, the patients interviewed were in a dependent position, in which they may not have felt free to speak their mind although the interviews were carried out by an independent researcher. This cannot be ruled out, but our experience is that many of these patients do not feel hesitant to tell us what they think of care providers and their involuntary admission. We accepted the critical notes they expressed in this study, and feel encouraged to stimulate free expression of opinion in our patients and participation in their treatment decisions. Second, the time between assessment and admission varied widely between the interviewed patients. The limited number of interviews available, and especially of repeated interviews of the same patients, did not permit studying any changes in treatment satisfaction and quality of life during admission. We therefore had to settle for correction of time since admission in the analyses. This time varied between 8 days and 3½ years. Finally, this study is cross-sectional and does not allow for causal inferences. Differences in attitude to admission, perceived coercion, therapeutic relationship, and perceived benefits may not only affect treatment satisfaction and quality of life, but may also be affected by these overall evaluations of treatment and living situation. The “determinants” studied provide more specific indications of which elements of treatment are evaluated positively or negatively by patients, and we interpret these as suggestions for treatment to focus on in order to try to improve patient outcomes.

## Conclusions

The current study shows that the most important aspects for treatment satisfaction and quality of life of dual-diagnosis patients admitted involuntary to long-term treatment are being listened to (being taken seriously) and experiencing improvements during treatment. These qualities reflect the goals of Shared Decision Making and Perceived Procedural Justice in treatment, and may be used to guide efforts to improve involuntary treatment. The study also corroborates earlier findings that even when treated involuntarily, patients might not hold particular negative views regarding their treatment.

## Data Availability Statement

The raw data supporting the conclusions of this article will be made available by the authors, without undue reservation.

## Ethics Statement

The protocol of this study was reviewed and approved by the Dutch Medical Ethical Committee for the Mental Health Services and judged to be in accordance with the Dutch Medical Research Involving Human Subjects Act and the Dutch Medical Treatment Agreement Act (Metc no: NL30019.097.09). Written informed consent for participation was not required for this study in accordance with the national legislation and the institutional requirements.

## Author Contributions

GV contributed to the study design, literature search, data acquisition, interpretation of results, responsible for manuscript writing, and revision. WD contributed to the study design, literature search, data acquisition, interpretation of results, and revision of the manuscript. RV was responsible for the study design, contributed to the literature search and interpretation of results, and revision of the manuscript. WM contributed to the design of the study and revised the manuscript critically for important intellectual content. GP contributed to literature search, interpretation of results, and revision of the manuscript. CM was responsible for the management of the study, contributed to the interpretation of results, and revision of the manuscript. All authors have read and approved the final manuscript.

## Conflict of Interest

The authors declare that the research was conducted in the absence of any commercial or financial relationships that could be construed as a potential conflict of interest.

## Publisher's Note

All claims expressed in this article are solely those of the authors and do not necessarily represent those of their affiliated organizations, or those of the publisher, the editors and the reviewers. Any product that may be evaluated in this article, or claim that may be made by its manufacturer, is not guaranteed or endorsed by the publisher.

## Abbreviations

SuRe: Sustainable Residence
SMI: Severe Mental Illness
PRO: Patient reported Outcome
TS: Treatment Satisfaction
SQOL: Subjective Quality of Life
CAT: Client Assessment of Treatment scale
MANSA: Manchester Short Assessment of Quality of Life
HAS: Helping Alliance Scale
BIS: Birchwood Insight Scale.
